# Moving toward a better understanding of the model bacterial predator *Bdellovibrio bacteriovorus*

**DOI:** 10.1099/mic.0.001380

**Published:** 2023-08-03

**Authors:** Simon G. Caulton, Andrew L. Lovering

**Affiliations:** ^1^​ School of Biosciences, University of Birmingham, Birmingham B15 2TT, UK

**Keywords:** Predatory Bacteria, Bdellovibrio, protein structure function

## Abstract

The bacterial predator *Bdellovibrio bacteriovorus* is a model for the wider phenomenon of bacteria:bacteria predation, and the specialization required to achieve a lifestyle dependent on prey consumption. *Bdellovibrio bacteriovorus* is able to recognize, enter and ultimately consume fellow Gram-negative bacteria, killing these prey from within their periplasmic space, and lysing the host at the end of the cycle. The classic phenotype-driven characterization (and observation of predation) has benefitted from an increased focus on molecular mechanisms and fluorescence microscopy and tomography, revealing new features of several of the lifecycle stages. Herein we summarize a selection of these advances and describe likely areas for exploration that will push the field toward a more complete understanding of this fascinating ‘two-cell’ system.

## Years of intrigue – classic early insights into predation

**Fig. 1.**
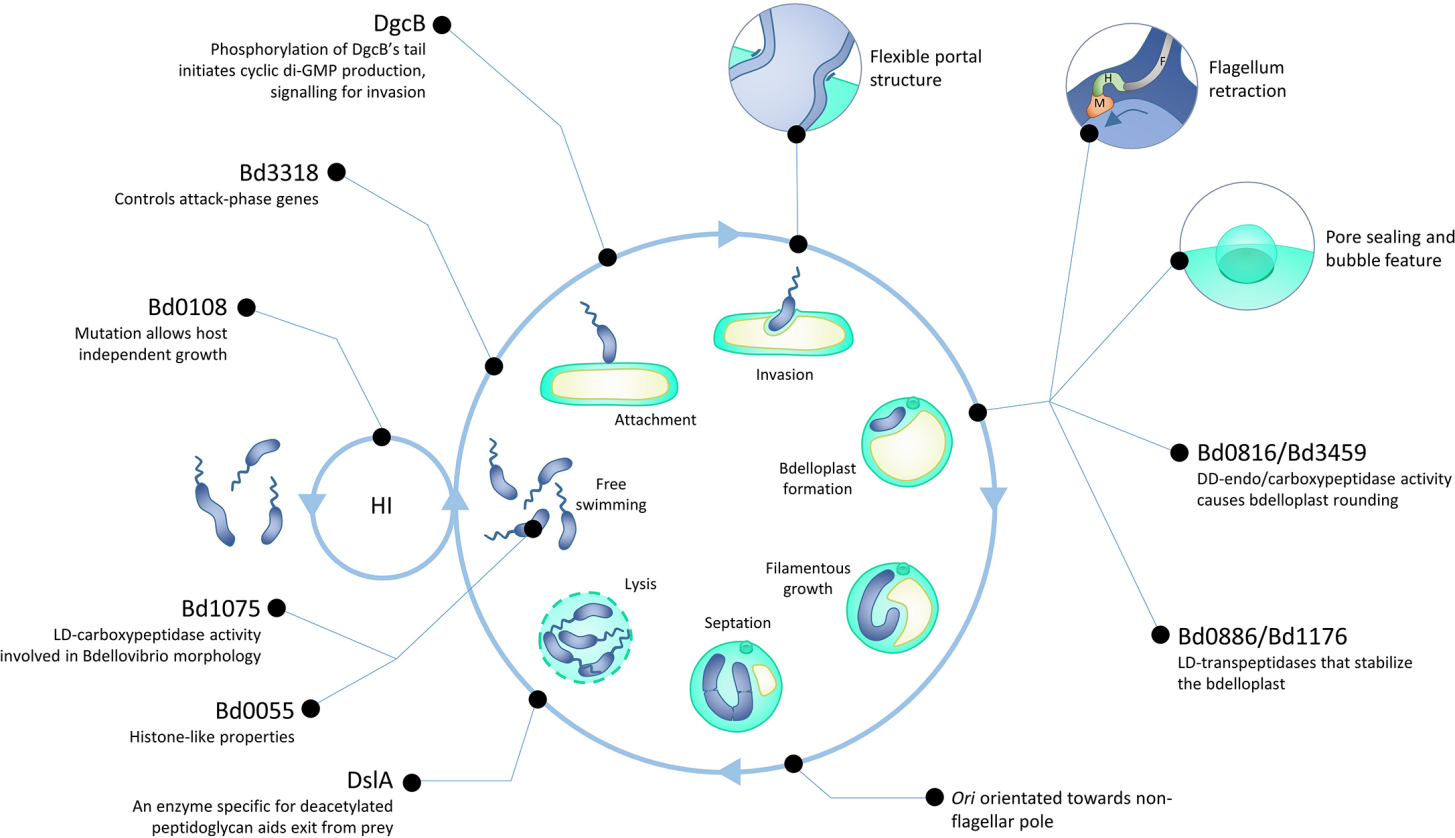
Stages of the *Bdellovibrio bacteriovorus* lifecycle, where predatory *Bdellovibrio* (darker blue) is shown invading and consuming a generic Gram-negative prey (lighter cyan and yellow). The role of specific named genes from the text are included at particular events/stages. The associated HI (host-independent) growth mode is also featured.

For those most familiar with ‘regular bacteria’, particularly chemoheterotrophs like *Escherichia coli *, personal discovery of the predatory bacterium *Bdellovibrio bacteriovorus* is frequently associated with delight and intrigue. To observe one bacterium enter, consume and rupture another (endoperiplasmic predation) often results in a series of ‘how?’ questions, framed by knowledge of more conventional bacterial physiology. Some of these questions are not limited to the uninitiated, leaving several major aspects of predation a source of active investigation in the field. The initial identification of *Bdellovibrio* was serendipitous, coming during a search for viruses from soil samples in which filter size allowed the relatively small predator through [1]. Subsequent experimentation revealed a specificity for Gram-negative prey cells, although the barrage of enzymes and lytic factors produced by *Bdellovibrio* retains some efficacy against Gram-positive cells also, though this might be considered an off-target effect [2]. Microscopic observations were followed by elegant electron microscopy (detailed in this wider review [3]), which established the principles behind the predatory lifecycle (Fig. 1). *Bdellovibrio* and like organisms (often abbreviated to BALOs), have a free-swimming attack phase during which they search for prey, and are among the fastest bacteria recorded. The search process has been hard to investigate, but hydrodynamic forces play a role [4] and identifiable chemotactic apparatus is encoded and used [5, 6], for maximal efficiency one would suspect ligands for this process to be absent/less prevalent in non-Gram-negatives and non-prey. *Bdellovibrio* then adheres to the prey cell and a recognition and signalling process occurs during which it may leave or progress to committed attachment. The predator then enters through the prey outer membrane, taking care to maintain the integrity of the cell being invaded; the point of entry is noticeably narrower than the predator cell width, a feature that tallies with *Bdellovibrio* having a relatively ‘soft’ and deformable membrane itself [7]. Having squeezed into the prey periplasm, the entry pore is resealed to avoid loss of nutrients, and (for rod-shaped prey) the invaded Bdelloplast structure changes to a rounded form. The microscopically apparent rounding event was later shown to result from prey cell peptidoglycan modifications that are used to serve as an occupancy signal that prevents other *Bdellovibrio* from wastefully invading a cell already being consumed [8]. The prey cell contents are then utilized by the predator, which grows as a filament, before dividing into a variable number of daughter cells, which may be odd or even – later beautifully demonstrated via use of ‘backlighting’ in a fluorescently-modified prey cell [9]. The prey cell is ruptured, and progeny escape to begin the cycle anew, a process that usually takes in the region of 4 h under laboratory conditions.

Without providing an exhaustive account, the earlier decades of *Bdellovibrio* research unearthed many points of interest, including: an ability to grow and divide outside of prey (Host-independent HI strains, licensed via mutation [10]); deacetylation of prey peptidoglycan to serve in part as a ‘me vs you’ marker during later stages of the lifecycle [11, 12]; breakage of the prey peptidoglycan-Braun lipoprotein link, followed by acylation of the wall using a lipid [13]; determination that the host cell inner membrane remains intact during predation but cytoplasmic and periplasmic pools equilibrate to some degree [14]; and transfer of a *Bdellovibrio* outer membrane protein into the prey inner membrane [15]. In this timeframe, other unusual *Bdellovibrio* strains were documented, including one that could stall/encyst within prey [16], and an epibiotic relative that remained attached during predation rather than invading [17].

## A genomic turning point for new studies

Using model strain HD100, Rendulic and co-workers sequenced the first *Bdellovibrio* genome in 2004 [18]. Key initial findings included a lack of gene transfer from prey; confirmation of an inability to synthesize certain amino acids and vitamins; an abundance of secreted proteins including proteases and nucleases; an association of the major HI-mutated gene with a type four pilus operon; and an appreciation that approximately one third of genes lacked any significant homology to genes of known function. Importantly, the first genome acted as a reference point for exploratory studies, enabling array-based identification of genes differentially regulated during invasion (‘the predatosome’, [19]) and RNA-seq identification of genes whose transcription is largely exclusive to either the attack phase or growth phase of the lifecycle [20]. Many invasion-associated genes formed part of the cryptic third outlined above, but importantly demonstrated a relative enrichment for genes with at least partial homology to known peptidoglycan modifying enzymes. Early unbiased transposon-based searches for genes essential to predation identified chemotaxis, flagellar and pili components [21] and also proteases, regulators and signalling proteins [22], alongside several cryptic functions; these have since been expanded to 100^+^ genes required for predative growth [23]. The majority of these candidates await mechanistic characterization.

Despite the relative uniqueness of *Bdellovibrio */BALOs, genome sequencing provided an ability to compare with other annotated genomes. A broad comparison of predators to non-predators by the Jurkevitch group found largely metabolic commonalities [24], presumably because (i) predators employ a shared ‘take not make’ strategy for the building blocks of biomolecules, and (ii) the differing killing modes of the predators compared in the study meant their predation genes were unique. *Bdellovibrio* is phylogenetically closest to the deltaproteobacterial wolfpack predator *Myxococcus xanthus *, which kills via contact and enzymes/antimicrobials [25]. Most importantly, similarity between *Bdellovibrio* and *Myxococcus* reveals a shared usage of gliding motility [26] and tight adherence (tad) pili [25], which though speculative form part of the genes required for predation [23]. In time, better characterization of other predators like the α-proteobacterial BALO *Micavibrio* [27], epibiotic *Vampirococcus lugosii* [28], and phagocytosis-like *Candidatus Uabimicrobium amorphum* [29] might reveal currently obscure thematic links between all styles of predation.

## Linking genes to predation phenomena

The genome sequence and follow-on -omics studies, through homology, provided putative functions that could be tested for a role in the predation lifecycle. Bdelloplast rounding was linked to two peptidoglycan dd-endopeptidases identified in the predatosome set [8, 19], alongside a co-expressed immunity protein that prevented predator self-modification and thus suicide [30]. Likewise, the peptidoglycan deacetylation originally observed by Thomashow and Rittenberg was conclusively linked to a predatosome lipoprotein [31], whose activity primes the prey cell wall for a late-acting deacetylation-specific enzyme that aids predator exit [12].

There have been several studies looking at the importance of type IV pili (TFP), Avidan and colleagues finding three type IVb flp subunits essential for predation [32], Evans *et al*. demonstrating essentiality of the major pilin pilA [33], and Mahmoud *et al*. demonstrating that antibodies against pilA delayed predation [34]. *Bdellovibrio* TFP are deployed at a single invasive pole (in contrast to the bipolar system of *M. xanthus *), where a signalling hub is subject to control by several factors including receptors for the ubiquitous bacterial second messenger cyclic-di-GMP [35]. Analysis of the genome predicts a relatively enriched role for cyclic-di-GMP signalling, borne out by knockout studies of the five *B. bacteriovorus* cyclic-di-GMP synthases, which reveal regulatory roles in invasion, HI growth and exit [36]. Interestingly, the exit process, where a synthase regulated escape from prey debris using gliding motility, was later shown to be a result of a hybrid enzyme that produced the related compound 3',3'-cyclic-GMP-AMP instead [37]. The importance of cyclic-di-GMP in several lifecycle stages led to a pulldown study that found 84 candidate binding proteins [38], inclusive of novel proteins and known cyclic-di-GMP signalling domains. These candidate proteins appear in several of the -omics studies above, and also studies looking into other aspects of *Bdellovibrio* biology, including the switch between a ‘resting’ state and active predation [39]. Nucleotide signalling in *Bdellovibrio* is undoubtedly complex, and discrete cyclic-di-GMP action potentials are controlled by a hydrolase responsive to the cyclic mononucleotide cAMP [40].

Other select *Bdellovibrio* phenomena and features that have been linked to particular genes include HI-growth being linked to mutations in the intrinsically disordered protein Bd0108 [10, 41], generation of predator curvature by the ld-carboxypeptidase Bd1075 [42], stabilization of the invaded cell by ld-transpeptidases Bd0886 and Bd1176 [43], control of attack phase genes by the flagellar sigma factor Bd3318 [20], and remarkably, nucleoid-organization by a first-in-class bacterial histone, Bd0055 [44, 45].

## Relevance in the era of amr

The prospect of using a natural antibacterial like *Bdellovibrio* against problematic bacteria, either in a bioremediation or therapeutic sense, is an interesting prospect that has been trialled and developed in several different settings. Early studies by Fratamico and Cooke in 1996 verified predation on stainless steel surfaces used in food processing [46], and for moderately similar purposes *Bdellovibrio* was found to guard against food spoilage by the mushroom pathogen *Pseudomonas tolaasii* [47]. Progression through a series of efficacy tests and infection models (reviewed herein [48]) has provided several proof-of-principle examples including: successful reduction of *Salmonella* numbers when orally-administered to chicks [49]; reduction of *Klebsiella* burden in a rat lung model [50]; no visible attachment to human cell lines [51]; and a visually-striking demonstration of *in vivo* predation in a zebrafish model of *Shigella* infection [52], where *Bdellovibrio* worked in tandem with the immune system to clear the pathogen. The acquisition and carriage of antimicrobial resistance genes by prey does not deleteriously effect predation by *Bdellovibrio* [53], although usage of the potential barrier of an S-layer can partly protect prey [54]. Strains susceptible to predation have not conclusively been shown to become resistant via mutation, although manual deletion of the OmpF porin affected predation kinetics (an effect particular to *E. coli* prey, not demonstrated to be a direct effect at present, [55]). Conversely, a loss-of-function screen identified mutation of secretion system genes as conveying predation susceptibility to a previously resistant *Acidovorax citrulli* [56]. One tantalising prospect would be to understand the factors behind prey recognition and ascertain whether a bespoke strain could be generated that had targeted specificity and left microbiota unscathed.

A putative therapeutic role, in the knowledge that many infections have a biofilm-related component, would take advantage of the ability of *Bdellovibrio* to be most efficient when searching and killing across a surface (as opposed to the hit-and-miss affair of planktonic collision). This mode of surface ‘scouting’ is enabled by gliding motility [57], deficits in which manifest in the aforementioned transposon studies to identify essential predation genes [21–23]. High-resolution microscopy has been used to map predation in a biofilm of *Vibrio cholerae *, demonstrating that *Bdellovibrio* can loosen biofilm architecture, although prey can generate some level of protection at higher cell densities [58].

## Observation at different scales

There have been considerable advances made in observing predatory features, from the microscopic to the atomic level. The original model for the staged lifecycle has recently been embellished from a variety of approaches, generating several surprises. The importance of prey cell peptidoglycan modification led Kuru *et al*. to image predation utilizing fluorescent d-amino acid reporters of transpeptidase activity [43]. These probes revealed that the entry pore is reinforced by a collar of peptidoglycan, which is then sealed shut – alongside prey cell reinforcement by ld-transpeptidases Bd0886/Bd1176, this highlights the importance of non-hydrolytic ‘niche formation’ events during predation. In terms of developmental biology, fluorescence microscopy has been used to establish that *Bdellovibrio* is able to stall and restart chromosome replication if prey resources are insufficient [59], and that asynchronous initiation of replication provides a mechanism to cope with both odd and even numbers of daughter cells [60]. The long-held requirement for filamentous growth and division has been challenged by observations with smaller *Proteus mirabilis* prey (< 2 µm), that reveal a remarkable shift to binary fission – this is accompanied by a requirement to remodel the old flagellar pole into a new invasion pole [61]. Additionally, progeny number can be conclusively linked to prey cell size [62], and the origin of replication is always located at the non-flagellated invasive pole [63].

Prior cryo-electron tomography of isolated *B. bacteriovorus* cells established aspects of *Bdellovibrio* physiology – deformable cells, condensed nucleoid, chemotaxis arrays, cryptic filaments and storage granules [7], but was unable to inform on any of the stages of predation. This has been supplemented by recent analysis of *Bdellovibrio* invading three types of prey – *V. cholerae, E. coli*, and *E. coli* minicells [64]. The ability to follow predation events is transformative, revealing for the first time that the predator retracts and likely recycles its own flagellum during invasion, disproving ideas that it is shed prior to entry. Alongside the rounding/occupancy discovery by Lerner *et al*., this finding provides another example of fitness and economy being key factors in predation, as can be gleaned from older biochemical studies that note *Bdellovibrio* conserves and directly utilizes the phosphate ester bonds of prey cell nucleotides [65]. An electron-dense attachment plaque spans from the predator outer membrane to the prey cell wall, next to rose-like complexes of undetermined composition. Other observable features include surface fibres distinct from pili, a protruding ‘bubble’ at the sealed entry pore, and a partially ordered hexagonal lattice of ribosomes on the nucleoid of fed late-stage cells [64]. Features not observed are also telling, with no evidence for type IV pili in providing the entry force to overcome the pushback from periplasmic pressure – the means by which the predator translocates inward into prey remains enigmatic.

**Fig. 2.**
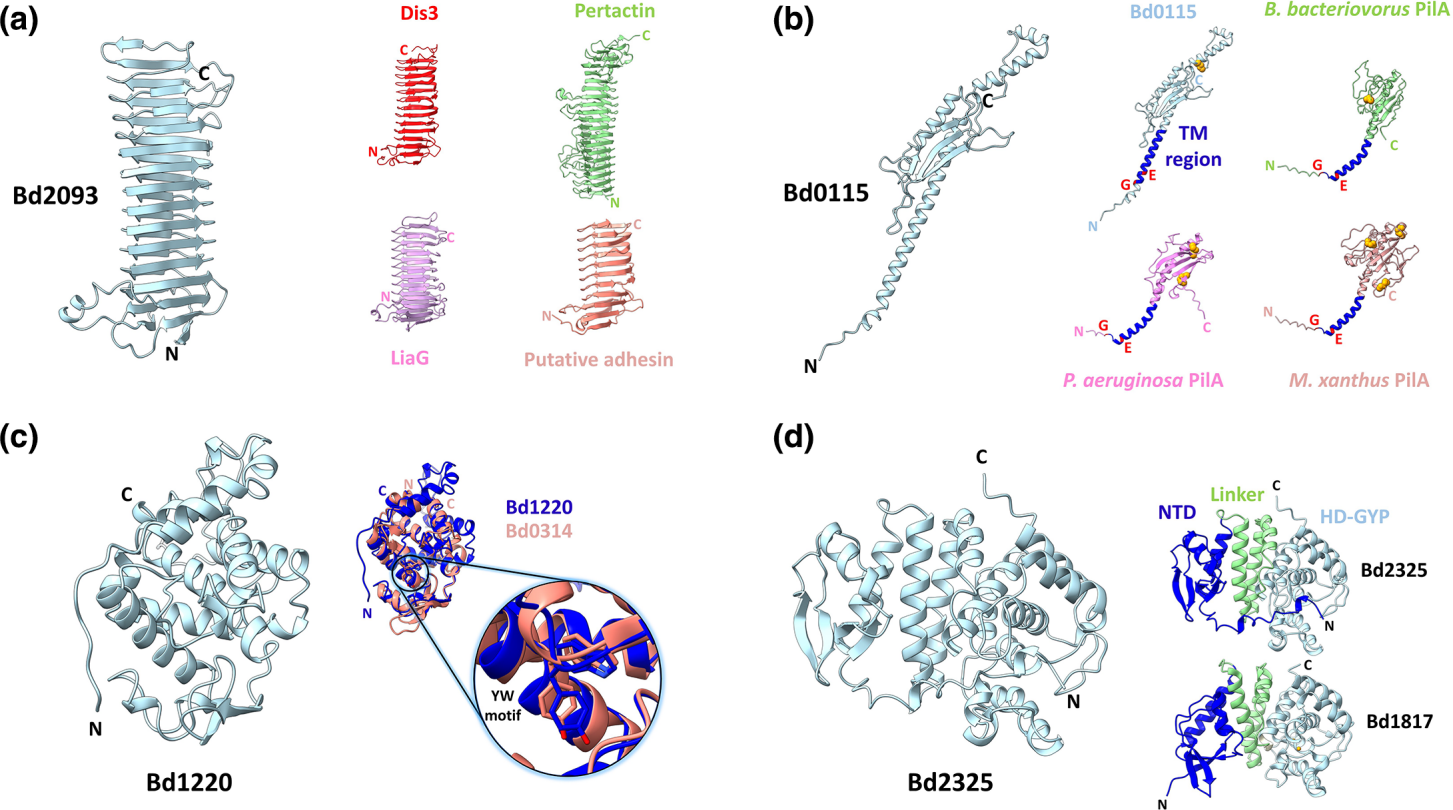
Usage of models to help suggest potential functions for cryptic *Bdellovibrio* genes. (**a**) Left, Alphafold model of the beta solenoid Bd2093 (Uniprot entry Q6MLC0, identified in Tudor *et al*. transposon study [22]). Right, similar protein structures (but disparate functions!), including the *Legionella pneumophila* Dot/Icm T4SS OMC component Dis3 (PDB code 7MUD); the *Bordetella pertussis* adhesin, pertactin (PDB code 1DAB); the *Bacillus subtilis* stress response protein LiaG (Alphafold model for Uniprot entry O32200); and a putative adhesin from *Bacteroides fragilis* (PDB code 3PET). (**b**) A more clear-cut equivalence: Left, Alphafold model of the pilin-like Bd0115 (Uniprot entry Q6MRG9). Right, Bd0115 and three selected pilin Alphafold models from *B. bacteriovorus* (Uniprot entry D3K481), *Myxococcus xanthus* (Uniprot entry Q59589) and *Pseudomonas aeruginosa* (Uniprot entry P17838). The Bd0115 model shows similarities to pilin structures, including the transmembrane (TM) region (blue) and the G-X-X-X-X-E pilin motif (Gly and Glu red), however it shows a C-terminal alpha-helical extension that is locked in place by a C-terminal disulphide bond (disulphide bonds shown as orange spheres). (**c**) Left, Alphafold model of the putative lysozyme Bd1220 (Uniprot entry Q6MNM6). Right, superposition of the Bd1220 model (Blue) with the identified exit enzyme Bd0314/DslA [12] (Salmon; PDB code 6TA9), with the well-overlaid YW motif of the active-site loop highlighted. This structural equivalence was missed in the original study which used sequence similarity to identify putative homologues. (**d**) Left, Alphafold model of the HD-GYP hydrolase domain containing protein Bd2325 (Uniprot entry Q6MKR0, part of the same transposon set [22] and later identified in a study on 3',3'-cyclic-GMP-AMP hydrolysis [71]). Right, comparison of Bd2325 with Bd1817 (PDB code 3TM8), another HD-GYP hydrolase from *B. bacteriovorus* [72]. Both proteins contain the HD-GYP hydrolase domain (light blue), helical linker domain (green) and N-terminal domain (NTD). The Bd2325 model shows a displacement of the NTD relative to the HD-GYP hydrolase domain, and also has an N-terminal extension that buries into the active-site of the HD-GYP hydrolase domain.

At the smallest scale, resolving the mechanism of individual proteins through structural studies, several lifecycle events can be explained. For example, the cyclic-di-GMP produced to initiate invasion is a result of the DgcB enzyme becoming activated when its own tail is phosphorylated, hence implicating kinase pathways at the onset of predation [66]. Identification of the phosphosensing forkhead domain as a key player is interesting, particularly given the potential for parallel activation of similar forkhead domains in *Bdellovibrio* type II secretion proteins and gliding motility regulators [18], which could potentially accompany invasion. At the other end of the cycle, a high resolution structure of the exit lysozyme DslA reveals precisely what adaptations allow activity on deacetylated prey material (generated at the start of predation, then ‘utilized’ during exit), and so revealing that this process of specialized modification and cleavage is actually shared with some non-predatory bacteria [12]. Such distant relationships between predators and non-predators are now made possible by sensitive structure:structure comparisons [67], which were not previously apparent with sequence-based methods. The dawn of AlphaFold models [68] is able to provide some clues to cryptic predation proteins, which will nevertheless require experimental confirmation and characterization. Examples of these are given in Fig. 2. Hypothesis generation via careful AlphaFold analysis may eventually assist the molecular understanding of several cryptic *Bdellovibrio* lifestyle events, and potentially enable identification of larger features/complexes observed in tomograms.

## Future prospects

The ranking/choice of new tools to aid *Bdellovibrio* research is subject to personal preference, but one area of undoubted use would be the introduction of a reliable system for inducible expression. For in-bdelloplast efficacy, the inducer would have to penetrate three membranes (and the bdelloplast membrane has different permeability characteristics to those of non-invaded cells, [14]). There has been a proof-of-principle in generating ‘controllable’ predators, that place the attack phase sigma factor mentioned above under riboswitch control [69]. Observations of predator behaviour would benefit from more precise synchronization of cultures and the development of robust fitness assays to compare wild-type strains to mutants; recently, efforts have been made towards automating comparative predation assays in a more user-friendly manner [70]. In a wider sense, we have much to learn about predator variation and how this correlates with environment and prey distribution – this will necessitate moving beyond the model HD100 strain.

The recent studies outlined herein, and a growing research community interested in mechanism and function, will undoubtedly lead to a better understanding of *Bdellovibrio* – the two-cell relationship of predator and prey having much to offer beyond that of predation alone.
